# Depletion of the Microbiome Alters the Recruitment of Neuronal Ensembles of Oxycodone Intoxication and Withdrawal

**DOI:** 10.1523/ENEURO.0312-19.2020

**Published:** 2020-05-21

**Authors:** Sierra Simpson, Adam Kimbrough, Brent Boomhower, Rio McLellan, Marcella Hughes, Kokila Shankar, Giordano de Guglielmo, Olivier George

**Affiliations:** 1 Department of Psychiatry, University of California San Diego, La Jolla, CA 92093,; 2 Department of Neuroscience, The Scripps Research Institute, La Jolla, CA 92037

**Keywords:** addiction, dependence, FOS, gut-brain axis, microbiome, opioids

## Abstract

Substance use disorders have a complex etiology. Genetics, the environment, and behavior all play a role in the initiation, escalation, and relapse of drug use. Recently, opioid use disorder has become a national health crisis. One aspect of opioid addiction that has yet to be fully examined is the effects of alterations of the microbiome and gut-brain axis signaling on central nervous system activity during opioid intoxication and withdrawal. The effect of microbiome depletion on the activation of neuronal ensembles was measured by detecting Fos-positive (Fos+) neuron activation during intoxication and withdrawal using a rat model of oxycodone dependence. Daily oxycodone administration (2 mg/kg) increased pain thresholds and increased Fos+ neurons in the basolateral amygdala (BLA) during intoxication, with a decrease in pain thresholds and increase in Fos+ neurons in the periaqueductal gray (PAG), central nucleus of the amygdala (CeA), locus coeruleus (LC), paraventricular nucleus of the thalamus (PVT), agranular insular cortex (AI), bed nucleus of the stria terminalis (BNST), and lateral habenula medial parvocellular region during withdrawal. Microbiome depletion produced widespread but region- and state-specific changes in neuronal ensemble activation. Oxycodone intoxication and withdrawal also increased functional connectivity among brain regions. Microbiome depletion resulted in a decorrelation of this functional network. These data indicate that microbiome depletion by antibiotics produces widespread changes in the recruitment of neuronal ensembles that are activated by oxycodone intoxication and withdrawal, suggesting that the gut microbiome may play a role in opioid use and dependence. Future studies are needed to better understand the molecular, neurobiological, and behavioral effects of microbiome depletion on addiction-like behaviors.

## Significance Statement

The United States is currently experiencing a national opioid crisis. Opioids alter the microbiome by reducing gut motility. They are often concurrently prescribed with antibiotics, which further reduces the richness and diversity of the resident microbiome. The gut-brain axis has recently emerged as a significant contributor to stress and behavioral alterations, but it has not been explored with regard to opioid use disorder. The present study found that depletion of the microbiome produced widespread region- and state-specific changes in neuronal ensemble activation. Importantly, neuronal ensembles that were altered by antibiotic depletion were in regions of the brain that are involved in opioid use disorder in both intoxication and withdrawal states. These results represent an important advance in our understanding of the impact of the gut-brain axis on neuronal recruitment in different drug states and how the microbiome may play a role in opioid use and dependence.

## Introduction

More than 2 million Americans currently suffer from substance use disorders that are related to prescription opioid pain relievers, such as oxycodone (CDC/NCHS, 2017). Every day, 3900 people initiate the non-medical use of prescription opioids, and 78 die from overdose (CDC/NCHS, 2017). This issue represents a significant health crisis, with high relapse potential and oftentimes a need for life-long opioid replacement therapy. Opioid use disorder is thought to be driven by a cycle of positively reinforcing behaviors and symptoms of negative affect that alter homeostatic mechanisms in the brain, causing tolerance, relapse, and dependence (Koob and Moal, 1997; Koob, 2008, 2009). The prolonged use of opioids is also associated with major side effects, including alterations of microbiome composition by reducing gut motility and inducing constipation, nausea, and vomiting, in addition to several other symptoms of opioid-induced bowel dysfunction (Leppert, 2012; Ketwaroo et al., 2013). Chronic opioid use has been shown to alter the gut microbiota in both humans and mice through the expansion of the Gram-positive bacteria Firmicutes and a reduction of *Bacteroides* (Vinolo et al., 2011; Morrison and Preston, 2016). Concurrent antibiotic and opioid treatment further exacerbates the reduction of α diversity (Meng et al., 2015; Le Bastard et al., 2018; Wang et al., 2018). A reduction of these phyla has been shown to reduce microbiome metabolites with important signaling capabilities, such as short-chain fatty acids (den Besten et al., 2013). However, the effects of microbiome depletion on brain regions that are activated by oxycodone intoxication and withdrawal are unknown. This is a critical gap in the literature because changes in brain activation levels that are caused by microbiome depletion may have consequences on neuroadaptations that are produced by chronic oxycodone use, potentially increasing abuse liability.

To test the hypothesis that changes in the microbiome affect the brain’s response to oxycodone intoxication and withdrawal, we investigated the effect of depletion of the microbiome using a non-absorbable antibiotic cocktail for two weeks (Kiraly et al., 2016) on the level of activation of brain regions that are recruited during intoxication and withdrawal using the immediate early gene c-*fos* as a measure of neuronal activity (Bullitt, 1990; Koya et al., 2012; Chung, 2015). We used a translationally relevant animal model of oxycodone dependence that is characterized by chronic daily injections of oxycodone (Wiebelhaus et al., 2016). Successful depletion of the microbiome was assessed by 16s RNA sequencing and downstream α diversity analysis using the Shannon diversity index and Choa1 index with caecal size as a secondary measure (Kiraly et al., 2016; Ge et al., 2017; Kennedy et al., 2018). Microbiome depletion was characterized by a significant decrease in both Bacteroidetes and Firmicutes. These phyla are estimated to constitute 80–90% of the resident microbiota (Brooks et al., 2003; Belheouane et al., 2017). Finally, we focused our neuronal ensemble analysis on brain regions that are known to be involved in oxycodone intoxication and withdrawal, including regions in the extended amygdala [e.g., basolateral amygdala (BLA), central nucleus of the amygdala (CeA), and bed nucleus of the stria terminalis (BNST)], brain regions that are involved in the regulation of stress and pain processing [e.g., locus coeruleus (LC), paraventricular nucleus of the thalamus (PVT), and periaqueductal gray (PAG)], and brain regions that are involved in craving/reward processing [e.g., agranular insular cortex (AI) and lateral habenula medial parvocellular part (LHb)].

## Materials and Methods

### Experimental design

Oxycodone dependence was initiated using a passive injection model, in which rats were injected subcutaneously with oxycodone (2 mg/kg) every 12 h for 5 d, two weeks after microbiome depletion or water treatment. The rats were subjected to microbiome depletion using a cocktail of non-absorbable antibiotics in their drinking water. Control rats were given regular drinking water. Prolonged oxycodone administration leads to tolerance and physical dependence, demonstrated by withdrawal symptoms on the cessation of drug administration. Withdrawal was precipitated at the end of the injection paradigm with a subcutaneous injection of naloxone (1 mg/kg) in the saline (SAL) group, withdrawal (WD) group, and antibiotic-treated withdrawal (WD+ABX) group. For the intoxication state, the rats were given a saline injection at the same time as the naloxone group. The OXY group included animals that were not treated with antibiotics in the intoxicated state, and the OXY+ABX group included animals that were treated with antibiotics in the intoxicated state. Withdrawal scores were taken following the naloxone injection to confirm the withdrawal state. von Frey pain threshold tests were performed 2 h following the oxycodone injection to confirm the intoxication state and oxycodone-induced analgesia. von Frey pain threshold tests were also performed following naloxone injections in the SAL, WD, and WD+ABX groups to confirm withdrawal-induced hyperalgesia. The rats were killed and perfused 90 min after the naloxone injection in the WD, WD+ABX, and SAL groups and after 90 min following the saline injection (matched with the naloxone injection time point) in the OXY and OXY+ABX groups. Brains were then cryopreserved and stained for Fos expression.

### Subjects

Adult male (*n *=* *22) and female (*n *=* *25) Sprague Dawley rats were housed in groups of three under a 12/12 h light/dark cycle (light on at 10 P.M.) in a humidity-controlled vivarium with *ad libitum* access to tap water and food pellets (PJ Noyes). All of the procedures were conducted in strict adherence to the National Institutes of Health Guide for the Care and Use of Laboratory Animals and were approved by the Institutional Animal Care and Use Committee of The Scripps Research Institute. At the time of testing, the subjects’ body weight ranged between 280 and 350 for females and 350–400 g for males.

### Drugs

Oxycodone (Sigma-Aldrich) was dissolved in 0.9% sodium chloride (Hospira) and administered subcutaneously (2 mg/kg) every 12 h. Naloxone (Sigma-Aldrich) was dissolved in 0.9% sodium chloride (Hospira) and administered subcutaneously (1 mg/kg). Antibiotic doses were given at the following concentrations in drinking water according to a previous study (Kiraly et al., 2016): bacitracin (0.5 mg/ml), neomycin (2 mg/ml), vancomycin (0.2 mg/ml), and pimaricin (1.2 μg/ml). These antibiotics were selected because they are absorbed in the intestine and have been previously reported to only have efficacy in depleting the microbiome when administered orally. The antibiotic mixture was changed every 2 d. The rats were weighed weekly to ensure the maintenance of body weight.

### Mechanical nociceptive von Frey test

Mechanical nociception, reflected by hindpaw withdrawal thresholds, was determined by an observer who was blind to the experimental groups using von Frey filaments that ranged from 3.63 to 281.838 g as previously reported (Dixon, 1965; Kononoff et al., 2018). A test session began after 10 min of habituation to the testing environment immediately following the subcutaneous injection of naloxone (WD, WD+ABX, and SAL groups) or saline (OXY and OXY+ABX groups). A series of von Frey filaments were applied from below the wire mesh to the central region of the plantar surface of the left and right hindpaws in ascending order, beginning with the smallest filament (3.63 g). The filament was applied until buckling occurred, and it remained in place for ∼2 s. A sharp withdrawal of the hindpaw indicated a positive response. The stimulus was incrementally increased until a positive response was observed and then decreased until a negative response was observed according to the statistical up-down method of Dixon (1965). The 50% paw withdrawal threshold was determined by the formula *Xf + kδ*, where *Xf* is the last von Frey filament applied, *k* is the Dixon value that corresponded to the response pattern, and *δ* is the mean difference between stimuli. Once the threshold was determined for the left hindpaw, the same testing procedure was repeated for the right hindpaw after 5 min. Paw withdrawal thresholds were recorded at baseline, during intoxication, and during precipitated withdrawal.

### Withdrawal score

To precipitate withdrawal, the rats were subcutaneously injected with naloxone and then placed in a Plexiglas observation chamber. The rats were observed for 30 min for signs of withdrawal by three independent observers who were blind to the experimental conditions. Withdrawal symptoms were categorized using a scale that was modified from (Gellert and Holtzman, 1978) and (Cicero et al., 2002). Graded signs of withdrawal were scored as the following: wet dog shakes (one or two shakes = 2; three or four shakes = 3; more than or equal to four shakes = 4) and escape attempts (two to four attempts = 1; five to nine attempts = 2; ≥10 attempts = 3). The following behaviors were measured per instance: abnormal posture/writhing, teeth chattering, ptosis (drooping eyelids), diarrhea, profuse salivation, urination, swallowing movements, and chromodacyorrhea (red tears). The total withdrawal score was calculated as the sum of all of the individual withdrawal scores. Immediately after withdrawal testing, all of the rats were returned to standard cages and given unrestricted access to food and water.

### Immunohistochemistry

The rats were injected with oxycodone (2 mg/kg) on the sixth day of the dosing schedule. Two hours later, the rats were injected with either saline (OXY and OXY+ABX groups) or 1 mg/kg naloxone (WD, WD+ABX, and SAL groups) to maintain intoxication or precipitate withdrawal, respectively. Brains were collected 90 min after the injection of naloxone or saline to produce both intoxicated and withdrawal groups. The rats were deeply anesthetized and perfused with 100 ml of PBS followed by 100 ml of 4% paraformaldehyde (PFA). Brains were postfixed in 4% PFA overnight and transferred to 30% sucrose in a PBS/0.1% azide solution at 4°C for 2 d. Brains were frozen in powdered dry ice for 15–30 s and then sectioned coronally at 40-μm thickness throughout the brain using a cryostat. Representative sections were quantified from the following regions at the following coordinates according to the (Paxinos and Watson, 2007) rat brain atlas: PVT (bregma −3.36), LHb (bregma −3.36), BLA (bregma −3.12), CeA (bregma −3.12), PAG (bregma −5.76), anterior agranular insula bregma (+3.00), BNST (bregma 0.00), and LC (bregma −9.16).

### 3,3’-diaminobenzidine (DAB) staining

The sections were washed in PBS for 10 min three times and then incubated in 1% H_2_O_2_/PBS for 20 min to quench endogenous peroxidase activity. The sections were then rinsed for 10 min three times in PBS and incubated in blocking solution (PBS + 0.3% Triton X-100, 1 mg/ml bovine serum albumin, and 5% normal donkey serum) for 1 h. The sections were then incubated for 24 h at room temperature with rabbit monoclonal anti-Fos antibody (Cell Signaling Technologies, catalog no. 2250, RRID: AB_2247211) diluted 1:1000 in PBS/0.5% Tween 20 and 5% normal donkey serum. Following incubation with the primary antibody, the sections were washed for 10 min three times in PBS and incubated for 2 h in undiluted rabbit ImmPress horseradish peroxidase reagent (Vector Laboratories, catalog no. MP-7451, RRID: 2631198). The sections were then washed in PBS for 10 min three times and then developed for 6 min in Vector peroxidase DAB substrate (Vector Laboratories, catalog no. SK-4100, RRID: 2336382) enhanced with nickel chloride. Following rinses in PBS for 10 min three times, the sections were mounted on Fisher Super Frost Plus slides, air dried, and coverslipped with PVA-DABCO (Sigma-Aldrich, catalog no. 10981). Following coverslipping, images were acquired using a Keyence BZX700 fluorescent microscope and analyzed using Fiji software.

### Imaging and quantification

Quantitative analysis to obtain unbiased estimates of the total number of Fos+ positive cell bodies was performed using Fiji software with the analyze particles module (Schindelin et al., 2012). Slides were imaged on a Keyence BZX700 slide scanner and stitched at 2× magnification. Three sections were bilaterally analyzed for each rat and then averaged per animal (counted as *n *=* *1). A total of four to six animals of each sex per group were analyzed. Cell numbers were normalized to the area of the region of interest.

### Connectivity analysis

Functional connections between brain regions were determined by calculating interregional Pearson correlations for each treatment (*n *=* *8–10/group, two-tailed *p *<* *0.05 for positive and negative connectivity). The statistical analyses were performed using Statistica and GraphPad Prism 7 software.

### 16s RNA sequencing

At each time point, approximately two fecal pellets were harvested and put directly into a cryovial, which was placed on powered dry ice. The pellets were then stored at −80°C until processing for sequencing. At the time of DNA extraction, feces were thawed and extracted with the Qiagen DNeasy powersoil kit. The samples are then amplified by PCR in triplicate and then pooled. The 16S V4 gene was amplified using universal primers (525F-806R). Each sample was normalized to 240 ng per sample and purified. After purification, the A260/A280 ratio of the final pool was recorded to ensure purity, with a tolerance range of 1.8–2.0. The barcoded amplicons from all of the samples were normalized, pooled to construct the sequencing library, and sequenced using an Illumina MiSeq Sequencer. The sequencing primers were the following: forward, TATGGTAATTGTGTGYCAGMGCCGCGGTAA and reverse, AGTCAGCCAGCCGGACTACNVGGGTWTCTAAT; and index sequence, AATGATACGGCGACCACCGAGATCTACACGCT. After sequencing, the raw files were prepared, filtered for quality, and demultiplexed. Operational taxonomic units (OTUs) were selected using open-reference OUT picking based on 97% sequence similarity to the Greengenes database. Taxonomy assignment and rarefaction were performed using Qiita with 15,000 reads per sample (Gonzalez et al., 2018). The α diversity was measured using both the Shannon diversity index and Chao1 index and compared between baseline and treatment conditions. Bray–Curtis dissimilarity clustering analysis was performed for principal component analysis (PCoA) to generate the biplot.

### Statistical analysis

The data are expressed as mean ± SEM. The von Frey test data were first analyzed using a mixed-model ANOVA, with group (OXY or WD) and treatment (water or ABX) as the between-subjects factor and time point (baseline or withdrawal/intoxication) as the within-subjects factor. For these measures, no difference was found between antibiotic-treated and water-treated animals. Therefore, the groups were pooled for the state (intoxication or withdrawal) factor. Follow-up Student–Newman–Keuls *post hoc* tests were performed to assess effects of group at each time point when a significant interaction was revealed. For withdrawal scores, microbiome assessments (Shannon diversity index, Chao1 index, Bacteroidetes Δ, Firmicutes Δ), caecum weights, and the recruitment of Fos+ neuronal ensembles, one-way ANOVA was performed, followed by the Student–Newman–Keuls *post hoc* test. No differences in withdrawal scores were found between antibiotic-treated animals and untreated animals. Therefore, the groups were pooled for the state (intoxication or withdrawal) factor and analyzed using one-way ANOVA, followed by the Student–Newman–Keuls *post hoc* test. The data were analyzed using either Statistica 7 software (correlational analysis) or GraphPad Prism 7 software (ANOVA). Values of *p *<* *0.05 were considered statistically significant. All of the statistical results are detailed in the statistical table (Table 1).

**Table 1:** Statistical table.

Figure	Data structure	Type of test	Statistical value	*p* value
2*A* von Frey pooled	Two factors (group and time point)	Two-way ANOVA	*F*_(2,44)_ = 41.0	<0.001
2*B* withdrawal score pooled	One factor (treatment)	One-way ANOVA Student–Newman–Keuls *post hoc* test	*F*_(2,44)_ = 123.3	<0.001
2*C* von Frey treatment	One factor (treatment)	One-way ANOVA Student–Newman–Keuls *post hoc* test	*F*_(3,34)_ = 37.95	<0.001
2*D* withdrawal score	One factor (treatment)	One-way ANOVA Student–Newman–Keuls *post hoc* test	*F*_(3,34)_ = 52.9	<0.001
3*A* Shannon baseline	One factor (treatment)	One-way ANOVA Student–Newman–Keuls *post hoc* test	*F*_(2,15)_ = 2.745	0.096
3*A* Shannon post-treatment	One factor (treatment)	One-way ANOVA Student–Newman–Keuls *post hoc* test	*F*_(2,15)_ = 87.15	<0.001
3*A* Chao1 baseline	One factor (treatment)	One-way ANOVA Student–Newman–Keuls *post hoc* test	*F*_(2,15)_ = 1.52	0.251
3*A* Chao1 posttreatment	One factor (treatment)	One-way ANOVA Student–Newman–Keuls *post hoc* test	*F*_(2,15)_ = 6.873	<0.008
Bacteroidetes post-treatment	One factor (treatment)	One-way ANOVA Student–Newman–Keuls *post hoc* test	*F*_(2,15)_ = 8,844	<0.003
Firmicutes post-treatment	One factor (treatment)	One-way ANOVA Student–Newman–Keuls *post hoc* test	*F*_(2,15)_ = 27.43	<0.001
3*F* caecum weight	One factor (treatment)	One-way ANOVA Student–Newman–Keuls *post hoc* test	*F*_(4,42)_ = 52.74	<0.001
4 (BLA)	One factor (treatment)	One-way ANOVA Student–Newman–Keuls *post hoc* test	*F*_(2,26)_ = 22.3	<0.001
4 (PAG)	One factor (treatment)	One-way ANOVA Student–Newman–Keuls *post hoc* test	*F*_(2,26)_ = 16.9	<0.001
4 (LC)	One factor (treatment)	One-way ANOVA Student–Newman–Keuls *post hoc* test	*F*_(2,26)_ = 25.2	<0.001
4 (CeA)	One factor (treatment)	One-way ANOVA Student–Newman–Keuls *post hoc* test	*F*_(2,26)_ = 73.97	<0.001
4 (PVT)	One factor (treatment)	One-way ANOVA Student–Newman–Keuls *post hoc* test	*F*_(2,26)_ = 23	<0.001
4 (AI)	One factor (treatment)	One-way ANOVA Student–Newman–Keuls *post hoc* test	*F*_(2,26)_ = 5.92	<0.001
4 (BNST)	One factor (treatment)	One-way ANOVA Student–Newman–Keuls *post hoc* test	*F*_(2,26)_ = 55.7	<0.001
4 (LHb)	One factor (treatment)	One-way ANOVA Student–Newman–Keuls *post hoc* test	*F*_(2,26)_ = 37.3	<0.001
5 (BLA)	One factor (treatment)	One-way ANOVA Student–Newman–Keuls *post hoc* test	*F*_(2,24)_ = 18.8	<0.001
5 (PAG)	One factor (treatment)	One-way ANOVA Student–Newman–Keuls *post hoc* test	*F*_(2,24)_ = 32.6	<0.001
5 (LC)	One factor (treatment)	One-way ANOVA Student–Newman–Keuls *post hoc* test	*F*_(2,24)_ = 6.79	<0.005
5 (CeA)	One factor (treatment)	One-way ANOVA Student–Newman–Keuls *post hoc* test	*F*_(2,24)_ = 5.13	<0.014
5 (PVT)	One factor (treatment)	One-way ANOVA Student–Newman–Keuls *post hoc* test	*F*_(2,24)_ = 1.18	<0.325
5 (AI)	One factor (treatment)	One-way ANOVA Student–Newman–Keuls *post hoc* test	*F*_(2,24)_ = 1.18	<0.324
5 (BNST)	One factor (treatment)	One-way ANOVA Student–Newman–Keuls *post hoc* test	*F*_(2,24)_ = 2.24	<0.129
5 (LHb)	One factor (treatment)	One-way ANOVA Student–Newman–Keuls *post hoc* test	*F*_(2,24)_ = 2.71	<0.87
6 (BLA)	One factor (treatment)	One-way ANOVA Student–Newman–Keuls *post hoc* test	*F*_(2,26)_ = 2.45	<0.106
6 (PAG)	One factor (treatment)	One-way ANOVA Student–Newman–Keuls *post hoc* test	*F*_(2,26)_ = 17.6	<0.001
6 (LC)	One factor (treatment)	One-way ANOVA Student–Newman–Keuls *post hoc* test	*F*_(2,26)_ = 17.7	<0.001
6 (CeA)	One factor (treatment)	One-way ANOVA Student–Newman–Keuls *post hoc* test	*F*_(2,26)_ = 17.9	<0.001
6 (PVT)	One factor (treatment)	One-way ANOVA Student–Newman–Keuls *post hoc* test	*F*_(2,26)_ = 24.1	<0.001
6 (AI)	One factor (treatment)	One-way ANOVA Student–Newman–Keuls *post hoc* test	*F*_(2,26)_ = 3.87	<0.034
6 (BNST)	One factor (treatment)	One-way ANOVA Student–Newman–Keuls *post hoc* test	*F*_(2,26)_ = 21.6	<0.001
6 (LHb)	One factor (treatment)	One-way ANOVA Student–Newman–Keuls *post hoc* test	*F*_(2,26)_ = 38.1	<0.001

## Results

### Repeated passive oxycodone injections induced intoxication, dependence, and withdrawal behaviors

**Figure 1.**
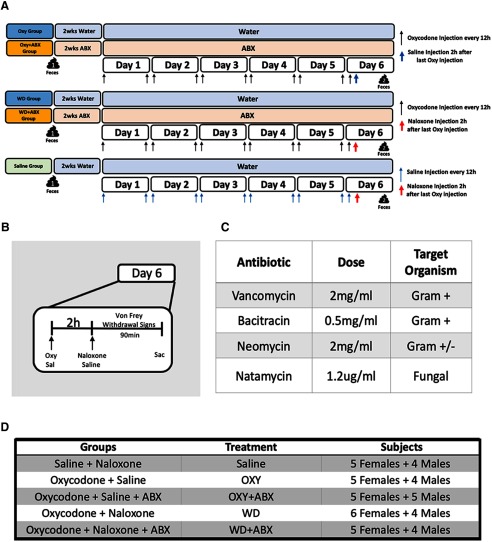
Experimental design. ***A***, The rats were made dependent on oxycodone using a passive injection model. The OXY group was not depleted with antibiotics and sacrificed during the intoxication state. The OXY+ABX group was depleted with antibiotics and sacrificed during the intoxication state. The WD group was not depleted with antibiotics and was sacrificed during naloxone-precipitated withdrawal. The WD+ABX group was sacrificed during the withdrawal state but was depleted with antibiotics. Feces for 16s RNA sequencing were taken at baseline (i.e., before antibiotic or drug exposure). A secondary time point was taken following the experimental paradigm. ***B***, Timeline for the behavioral tests to confirm the intoxication or withdrawal state (withdrawal scoring and von Frey test). ***C***, For depletion of the microbiome, the rats were exposed to a cocktail of both Gram-negative and Gram-positive antibiotics in drinking water for two weeks before the initiation of dependence. ***D***, Experimental groups, including both sexes.

Before initiating dependence, the rats (*n *=* *47; 22 males, 25 females) were given either water only or water that was mixed with a non-absorbable antibiotic cocktail for two weeks to deplete a significant portion of the microbiome as previously reported (Kiraly et al., 2016). Significant perturbation of the innate microbiome is necessary to reduce the resident richness and diversity of the microbial milieu (Reikvam et al., 2011). The rats were then given twice-daily injections of either oxycodone (2 mg/kg for 5 d) to induce dependence or saline as a control. On the sixth day, the rats received a naloxone injection (1 mg/kg) to precipitate withdrawal or a saline injection to maintain the state of intoxication. All of the antibiotic-treated groups were maintained on the treatment throughout the entire testing period. For a diagram of the experimental design, see Figure 1*A*. A behavioral testing timeline for the final day is presented in Figure 1*B*.

**Figure 2.**
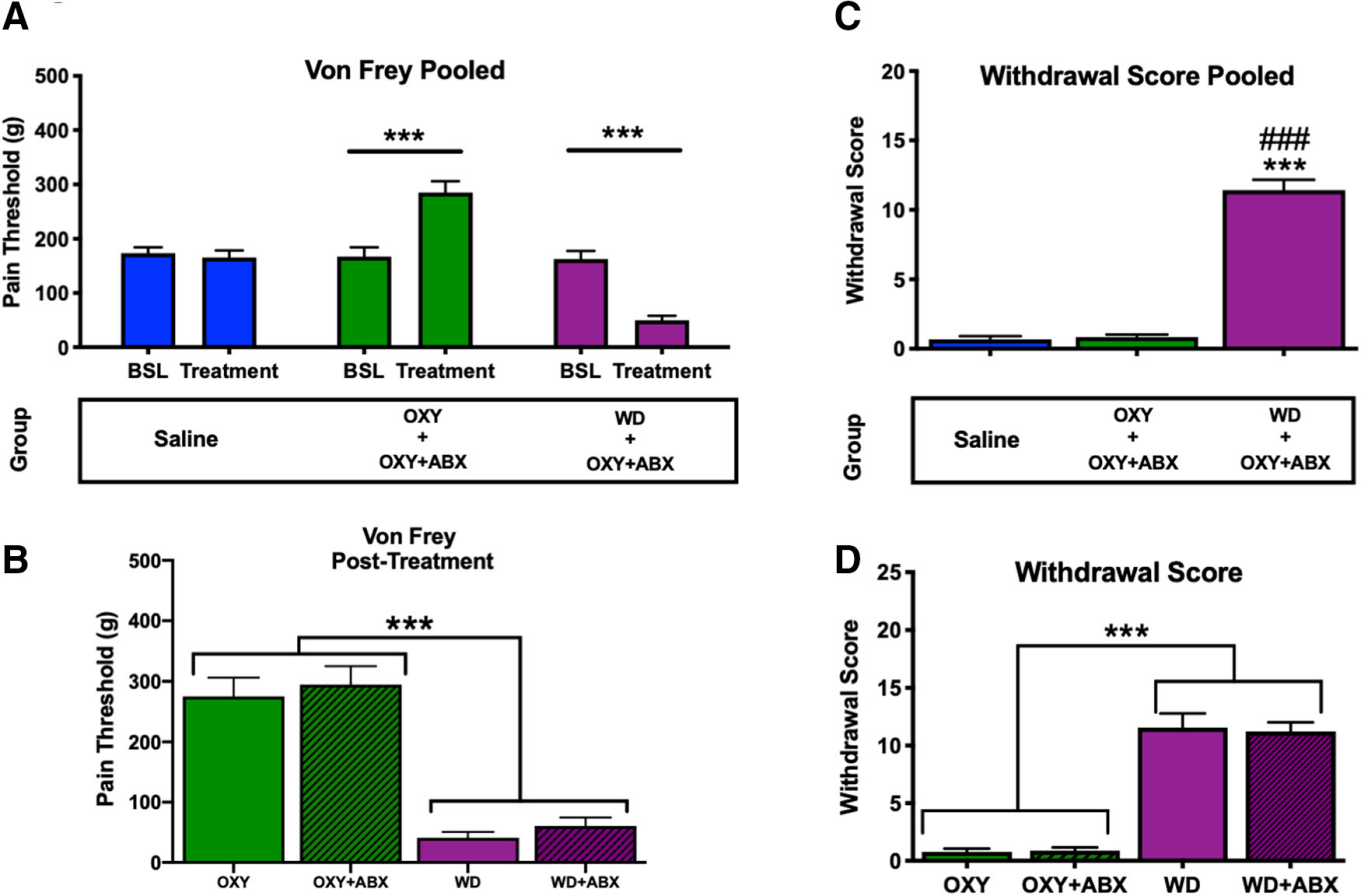
Validation of precipitated withdrawal and intoxication in passively injected rats. ***A***, ***B***, The von Frey test was used to confirm that oxycodone withdrawal decreased pain thresholds and oxycodone intoxication increased pain thresholds. No significant difference in pain thresholds was found between the antibiotic-treated and untreated groups. Therefore, each state was pooled. Intoxicated animals exhibited higher pain thresholds compared with the saline and withdrawal groups. The withdrawal group exhibited lower pain thresholds compared with the saline group (***p <* *0.001) and intoxication group (###*p *<* *0.001). ***C***, The total withdrawal score was calculated as the sum of all individual withdrawal scores. No difference in withdrawal scores was observed between antibiotic-depleted animals and untreated animals, so each state was pooled (***D***). The withdrawal animals exhibited higher withdrawal scores compared with intoxicated animals (*p *<* *0.001).

Paw withdrawal thresholds were examined using the von Frey test to evaluate mechanical nociception (OXY and OXY+ABX groups) and withdrawal-induced hyperalgesia/allodynia (WD, WD+ABX, and SAL groups; Dixon, 1965; Kononoff et al., 2018). Intoxicated animals had significantly higher von Frey scores compared with baseline scores, and animals in withdrawal had significantly lower von Frey scores compared with baseline scores and intoxicated animals (*F*_(2,44)_ = 41.0, *p *<* *0.001; Fig. 2*A*). The one-way ANOVA revealed that at the intoxication/withdrawal time point, all of the groups significantly differed from one another, such that intoxicated animals had significantly higher von Frey scores and withdrawal animals had significantly lower von Frey scores compared with all of the other groups (all *p *<* *0.001; Fig. 2*A*,*B*).

Animals that underwent withdrawal had higher withdrawal scores compared with intoxicated animals, regardless of treatment (*F*_(2,44)_ = 123.3, *p *<* *0.001). We observed a significant main effect of group, in which animals in the withdrawal condition had significantly higher withdrawal scores compared with both saline and intoxicated animals (both *p *<* *0.001). No significant difference in withdrawal scores was found between saline and intoxicated animals (*p *>* *0.99).

### Antibiotic depletion of the microbiome decreased α-diversity and increased caecal size

**Figure 3.**
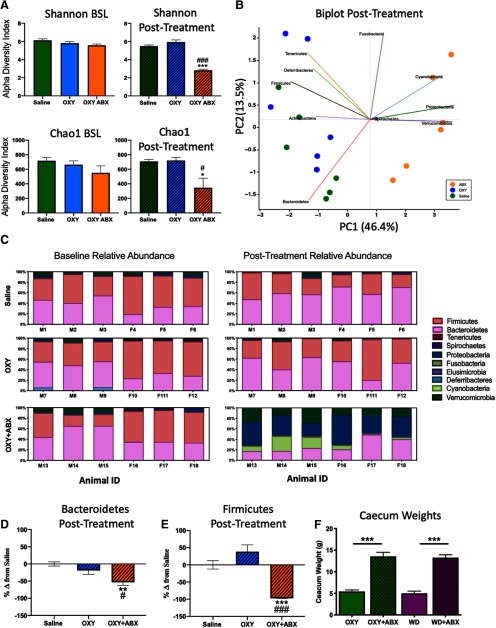
Antibiotic depletion reduces α-diversity. ***A***, Antibiotic depletion was measured using both the Shannon diversity index and Choa1 index. No difference in either index was found between groups for the initial baseline (BSL) time point. The final time point was taken following both antibiotic depletion and the final oxycodone exposure (posttreatment). In the posttreatment conditions, the OXY+ABX group exhibited a significant decrease in α diversity compared with both the SAL and OXY groups, indicated by the Shannon diversity index (*p *<* *0.001) and Chao1 index (*p *<* *0.008). ***B***, A biplot of the PCoA by Bray–Curtis similarity clustering indicated that the SAL and OXY groups clustered together, whereas the antibiotic-treated animals clustered separately. ***C***, Taxonomic plots that show the relative abundance of phyla across different groups at BSL and posttreatment. ***D***, ***E***, The OXY+ABX group exhibited a significant decrease in both Bacteroidetes (*p < *0.003) and Firmicutes (*p < *0.001) at the phylum level. Each animal is represented at both timepoints at the phylum level in the relative abundance plots. ***F***, Caecal weights were measured at the time of killing. A significant increase in caecal weights was observed in antibiotic-treated rats (*p *<* *0.001); *p <* *0.05, **p <* *0.002, ***p <* *0.001, significant difference from saline; #*p *<* *0.05, ##*p *<* *0.002, ###*p *<* *0.001 significant difference from OXY group.

To confirm that the microbiome was depleted by antibiotics, we assessed α-diversity of the samples based on the Shannon diversity index and Chao1 index. The Chao1 index qualitatively measures α-diversity but gives more weight to rare species. The Shannon diversity index accounts for both richness and evenness to determine α diversity. The one-way ANOVA revealed that antibiotic treatment significant decreased α-diversity (*F*_(2,15)_ = 87.15, *p *<* *0.001; Fig. 3*A*). The Student–Newman–Keuls *post hoc* test revealed no difference in the Shannon diversity index between the SAL and OXY groups (*p *<* *0.220), whereas the Shannon diversity index significantly decreased in the OXY+ABX compared with both the water group (*p *<* *0.001) and OXY group (*p *<* *0.001). Similarly, the Chao1 test revealed a decrease in α diversity in antibiotic-treated animals (*F*_(2,15)_ = 6.873, *p *<* *0.008). The Student–Newman–Keuls *post hoc* test showed no difference in α-diversity between SAL and OXY groups (*p *<* *0.993), whereas α-diversity decreased in the OXY+ABX group compared with both the SAL group (*p *<* *0.017) and OXY group (*p *<* *0.014). To ensure the absence of initial differences between groups, we compared the groups at baseline (i.e., before antibiotic or drug exposure). No difference in either Shannon diversity index (*F*_(2,15)_ = 2.745, *p *<* *0.096) or Choa1 index (*F*_(2,15)_ = 1.52, *p *<* *0.251) was observed (Fig. 3*A*).

A PCoA of fecal samples was performed to compare the SAL, OXY, and OXY+ABX groups. The SAL and OXY groups clustered together, whereas the OXY+ABX group clustered separately (Fig. 3*B*). At the phylum level, Bacteroidetes and Firmicutes were the most abundant under baseline conditions, accounting for 40.33% and 50.30% of the resident microbiota, respectively (Fig. 3*C*). After treatment, a decrease in Bacteroidetes was observed in the OXY group (−19.01%) and OXY+ABX group (−53.44%) compared with the matched SAL group. A decrease in Firmicutes was observed after treatment in the OXY+ABX group (−97.3%), whereas the OXY group exhibited an increase in Firmicutes following oxycodone exposure (38.5%) compared with the matched saline group. The OXY+ABX group exhibited increases in Cyanobacteria, Proteobacteria, and Verrucomicrobia compared with the SAL and OXY groups (Fig. 3*C*).

The shift in microbial communities was further analyzed by measuring changes in Bacteroidetes (Fig. 3*D*) and Firmicutes (Fig. 3*E*) relative to the matched saline group after treatment. Bacteroidetes significantly decreased following antibiotic treatment (*F*_(2,15)_ = 8.844, *p *<* *0.003). The Student–Newman–Keuls *post hoc* test revealed a significant decrease in Bacteroidetes in the OXY+ABX group compared with the water group (*p *<* *0.002) and the OXY group (*p *<* *0.03). No difference in Bacteroidetes was found between the OXY and SAL groups (*p *>* *0.05). A similar decrease in Firmicutes was observed after antibiotic treatment (*F*_(2,15)_ = 27.43, *p *<* *0.003). The Student–Newman–Keuls *post hoc* test revealed a significant decrease in Firmicutes in the OXY+ABX group compared with the SAL group (*p *<* *0.001) and the OXY group (*p *<* *0.001). No difference in Firmicutes was found between the OXY and SAL groups (*p *>* *0.05).

As previously reported, gnotobiotic mice and mice with antibiotic depletion exhibited an increase in caecal size. In the present study, animals that received antibiotic treatment had significantly heavier caeca weights compared with animals that did not receive antibiotic treatment, regardless of whether they were in intoxicated or withdrawal conditions (*F*_(3,34)_ = 55.34, *p *<* *0.001; Fig. 2*F*). No difference in caecal weight was observed among OXY or WD group compared with saline animals under water treatment conditions (*p *>* *0.73).

### Characterization of the recruitment of Fos+ neuronal ensembles of oxycodone intoxication and withdrawal

#### Basolateral Amygdala

The ANOVA revealed a significant effect of group on the number of recruited Fos+ neurons (*F*_(2,26)_ = 22.3, *p *<* *0.001). The Student–Newman–Keuls *post hoc* test revealed significantly fewer Fos+ neurons in the SAL group compared with the OXY group. The WD group exhibited a significant decrease in Fos+ neurons compared with the OXY group but no difference from the SAL group.

#### Central Amygdala

The ANOVA revealed a significant effect of group on the number of recruited Fos+ neurons (*F*_(2,26)_ = 73.97, *p *<* *0.001). The Student–Newman–Keuls *post hoc* test revealed no difference between the SAL and OXY groups but a significant difference between the SAL and WD groups and between the OXY and WD groups.

#### Locus Coeruleus

The ANOVA revealed a significant effect of group on the number of recruited Fos+ neurons (*F*_(2,26)_ = 25.2, *p *<* *0.001). The Student–Newman–Keuls *post hoc* test revealed a significant difference between the SAL group and the WD and WD+ABX groups and between the OXY group and WD and WD+ABX groups.

#### Periaqueductal Gray

The ANOVA revealed a significant effect of group on the number of recruited Fos+ neurons (*F*_(2,26)_ = 16.9, *p *<* *0.001). The Student–Newman–Keuls *post hoc* test revealed a significant increase in Fos+ neurons in the WD group compared with the SAL and OXY groups.

#### Lateral Habenula

The ANOVA revealed a significant effect of group on the number of recruited Fos+ neurons (*F*_(2,26)_ = 37.3, *p *<* *0.001). The Student–Newman–Keuls *post hoc* test revealed a significant increase in Fos+ neurons in the WD group compared with the SAL and OXY groups. No difference in the number of Fos+ neurons was found between the SAL and OXY groups.

#### Periventricular thalamic nucleus

The ANOVA revealed a significant effect of group on the number of recruited Fos+ neurons (*F*_(2,26)_ = 23, *p *<* *0.001). The Student–Newman–Keuls *post hoc* test revealed a significant increase in Fos+ positive neurons in the WD group compared with the SAL and OXY groups. No difference in the number of Fos+ neurons was found between the SAL and OXY groups.

#### Bed nucleus of the stria terminalis

The ANOVA revealed a significant effect of group on the number of recruited Fos+ neurons (*F*_(2,26)_ = 55.7, *p *<* *0.001). The Student–Newman–Keuls *post hoc* test revealed a significant increase in the number of Fos+ neurons in the WD group compared with the SAL and OXY groups. No significant difference in the number of Fos+ neurons was found between the SAL and OXY groups.

#### Anterior insula

The ANOVA revealed a significant effect of group on the number of recruited Fos+ neurons (*F*_(2,26)_ = 5.92, *p *=* *0.008). The Student–Newman–Keuls *post hoc* test revealed a significant increase in the number of Fos+ neurons in the WD group compared with the SAL and OXY groups.

### Characterization of the effect of antibiotic depletion on the recruitment of Fos+ neuronal ensembles of oxycodone intoxication and withdrawal

#### Basolateral Amygdala

**Figure 4.**
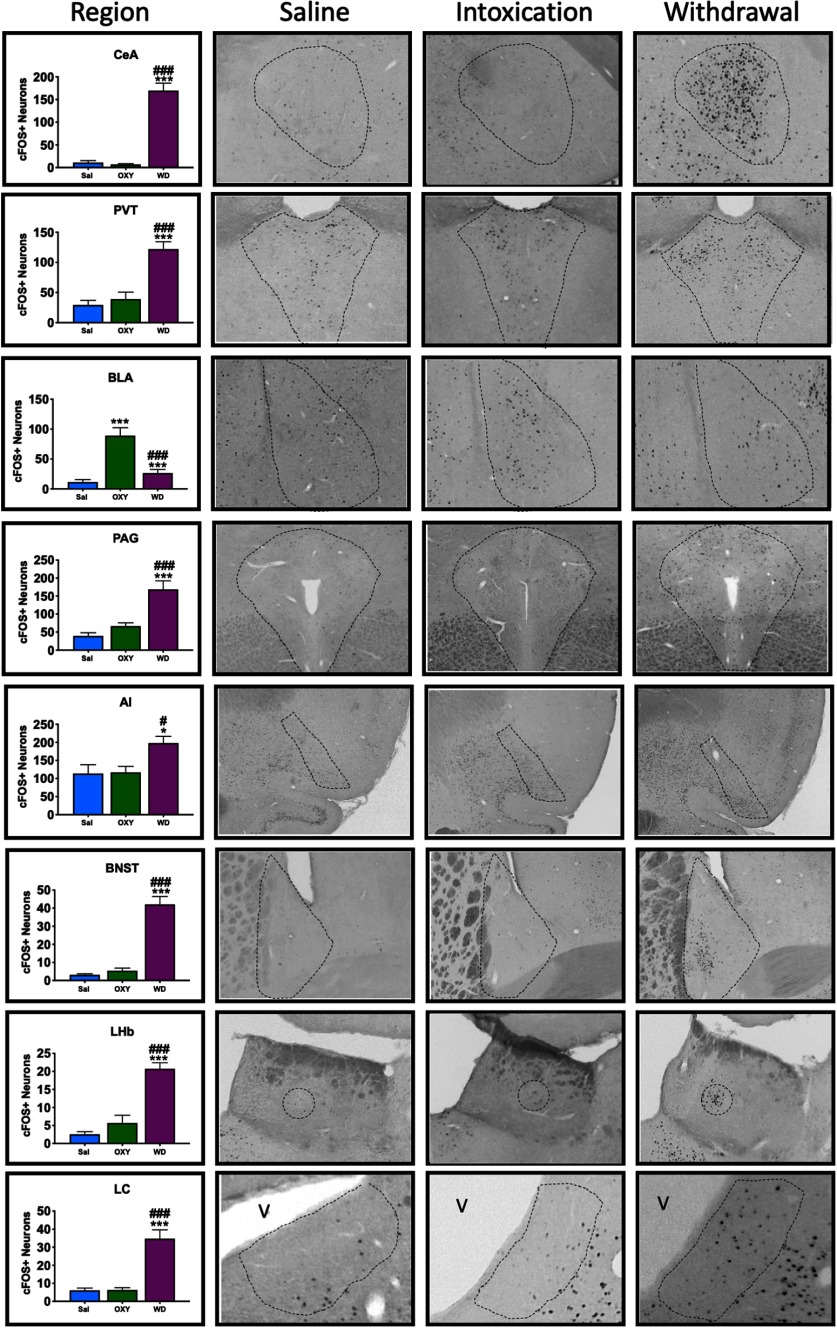
Quantification of Fos+ neuronal ensembles during oxycodone intoxication and withdrawal. Ensembles that were recruited during both intoxication and withdrawal were quantified by counting Fos+ neurons that were active 90 min before killing. Saline, saline + naloxone group; OXY, oxycodone + saline; WD, oxycodone + naloxone. Coronal slices (40 μM) were stained for Fos and then visualized using DAB enhanced with nickel to obtain representative images. The representative regions are outlined with dashes; *p <* *0.05, **p <* *0.002, ***p <* *0.001, significant difference from saline; #*p *<* *0.05, ##*p *<* *0.002, ###*p *<* *0.001 significant difference from OXY group.

**Figure 5.**
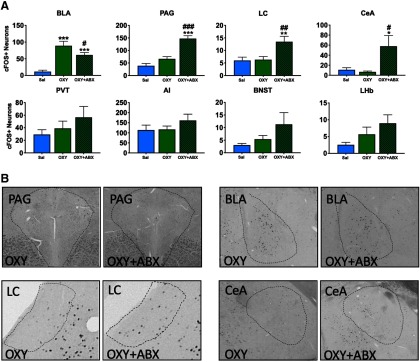
Quantification of Fos+ neuronal ensembles during oxycodone intoxication with antibiotic treatment. ***A***, Differences in the number of Fos+ neurons were evaluated during oxycodone intoxication between antibiotic-treated and untreated rats. The regions that were altered during intoxication are shown in green (OXY) and green with black stripes (OXY+ABX). Coronal slices (40 μM) were stained for Fos and then visualized using DAB enhanced with nickel to obtain representative images. Representative regions are outlined with dashes. ***B***, Example images of each region that was altered by antibiotic depletion; *p <* *0.05, **p <* *0.002, ***p <* *0.001, significant difference from saline; #*p *<* *0.05, ##*p *<* *0.002, ###*p *<* *0.001 significant difference from OXY group.

**Figure 6.**
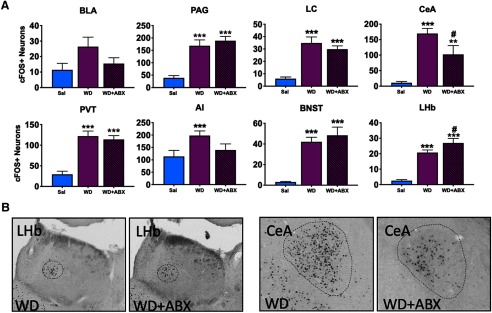
Quantification of Fos+ neuronal ensembles during oxycodone withdrawal with antibiotic treatment. ***A***, Differences in the number of Fos+ neurons were evaluated during oxycodone withdrawal between antibiotic-treated and untreated rats. The regions that were altered during withdrawal are represented in purple (WD) and purple with black stripes (WD+ABX). ***B***, Example images of each region that was altered by antibiotic depletion; *p <* *0.05, **p <* *0.002, ***p <* *0.001, significant difference from saline; #*p *<* *0.05, ##*p *<* *0.002, ###*p *<* *0.001 significant difference from non-depleted withdrawal group (WD).

The ANOVA revealed a significant effect of treatment on the number of recruited Fos+ neurons in the OXY group (*F*_(2,24)_ = 18.8, *p *<* *0.001; Fig. 5*A*). The Student–Newman–Keuls *post hoc* test revealed a significant decrease in the number of Fos+ neurons in the OXY group compared with the OXY+ABX group. Example DAB images are shown in Figure 5*B*, example images of the untreated, matched OXY group are repeated from Figure 4 for comparisons to the antibiotic-treated groups. There was no significant difference in the number of Fos+ neurons in the withdrawal group (WD and WD+ABX; *F*_(2,26)_ = 2.45, *p *<* *0.106; Fig. 6*A*).

#### Central Amygdala

The ANOVA revealed a significant effect of treatment on the number of recruited Fos+ neurons in the OXY group (*F*_(2,24)_ = 5.13, *p *<* *0.014) and WD group (*F*_(2,26)_ = 17.9, *p *<* *0.001). The Student–Newman–Keuls *post hoc* test revealed a significant decrease in the number of Fos+ neurons in the WD group compared with the WD+ABX group and a significant increase in the number of Fos+ neurons in the OXY group compared with the OXY+ABX group. Example DAB images are shown in Figures 5*B*, 6*B*. Example images of untreated, matched groups are repeated from Figure 4 for comparisons to the antibiotic-treated groups.

#### Locus Coeruleus

The ANOVA revealed a significant effect of treatment on the number of recruited Fos+ neurons in the OXY group (*F*_(2,24)_ = 6.79, *p *<* *0.005) and WD group (*F*_(2,26)_ = 17.7, *p *<* *0.001). The Student–Newman–Keuls *post hoc* test revealed a significant increase in the number of Fos+ neurons in the OXY+ABX group compared with the OXY group. Example DAB images are shown in Figure 5*B*. Example images from the OXY group are repeated from Figure 4 for comparisons to the antibiotic-treated groups. A significant difference in the number of Fos+ neurons was found between the SAL, OXY+ABX, WD, and WD+ABX groups.

#### Periaqueductal Gray

The ANOVA revealed a significant effect of treatment on the number of recruited Fos+ neurons in the OXY group (*F*_(2,24)_ = 32.6, *p *<* *0.001) and WD group (*F*_(2,26)_ = 17.6, *p *<* *0.001). The Student–Newman–Keuls *post hoc* test revealed a significant increase in the number of Fos+ neurons in the OXY+ABX group compared with the OXY group. Example DAB images are shown in Figure 5*B*. Example images from the OXY group are repeated from Figure 4 for comparisons to the antibiotic-treated groups. The SAL group exhibited a significant decrease in Fos+ neurons compared with all of the other groups. No significant difference in the number of Fos+ neurons was found between the WD and WD+ABX groups.

#### Lateral Habenula

The ANOVA revealed no effect of treatment on the number of recruited Fos+ neurons in the OXY group (*F*_(2,24)_ = 2.71, *p *<* *0.87) but a significant effect of treatment in the WD group (*F*_(2,26)_ = 38.1, *p *<* *0.001). The Student–Newman–Keuls *post hoc* test revealed a significant increase in the number of Fos+ neurons in the WD group compared with the WD+ABX group. Example DAB images are shown in Figure 6*B*. Example images from the WD group are repeated from Figure 4 for comparison. No difference in the number of Fos+ neurons was found between the SAL, OXY, and OXY+ABX groups (Fig. 5). A significant increase in the number of Fos+ neurons was observed in the WD and WD+ABX groups compared with the saline group.

#### Periventricular thalamic nucleus

The ANOVA revealed no effect of treatment on the number of recruited Fos+ neurons in the OXY group (*F*_(2,24)_ = 1.18, *p *<* *0.325) but a significant effect of treatment in the WD group (*F*_(2,26)_ = 24.1, *p *<* *0.001). The Student–Newman–Keuls *post hoc* test revealed a significant decrease in the number of Fos+ neurons in the SAL group compared with the WD and WD+ABX groups (Fig. 6*A*). No difference in the number of Fos+ neurons was found between SAL, OXY, and the OXY+ABX groups (Fig. 5*A*).

#### Bed nucleus of the stria terminalis

The ANOVA revealed no effect of treatment on the number of recruited Fos+ neurons in the OXY group (*F*_(2,24)_ = 2.24, *p *<* *0.129) but a significant effect of treatment in the WD group (*F*_(2,26)_ = 21.6, *p *<* *0.001). The Student–Newman–Keuls *post hoc* revealed no difference in the number of Fos+ neurons between the SAL, OXY, and OXY+ABX groups (Fig. 5*A*). The SAL group exhibited a significant decrease in the number of Fos+ neurons compared with the WD and WD+ABX groups (Fig. 6*A*).

#### Anterior insula

The ANOVA revealed no effect of treatment on the number of recruited Fos+ neurons in the OXY group (*F*_(2,24)_ =1.18, *p *=* *0.324) and WD group (*F*_(2,26)_ = 3.87, *p *=* *0.034). The Student–Newman–Keuls *post hoc* test revealed a significant increase in the number of Fos+ neurons in the WD group compared with the SAL group. There was no effect of antibiotics.

### Microbiome depletion induced alterations of intoxication network connectivity

**Figure 7.**
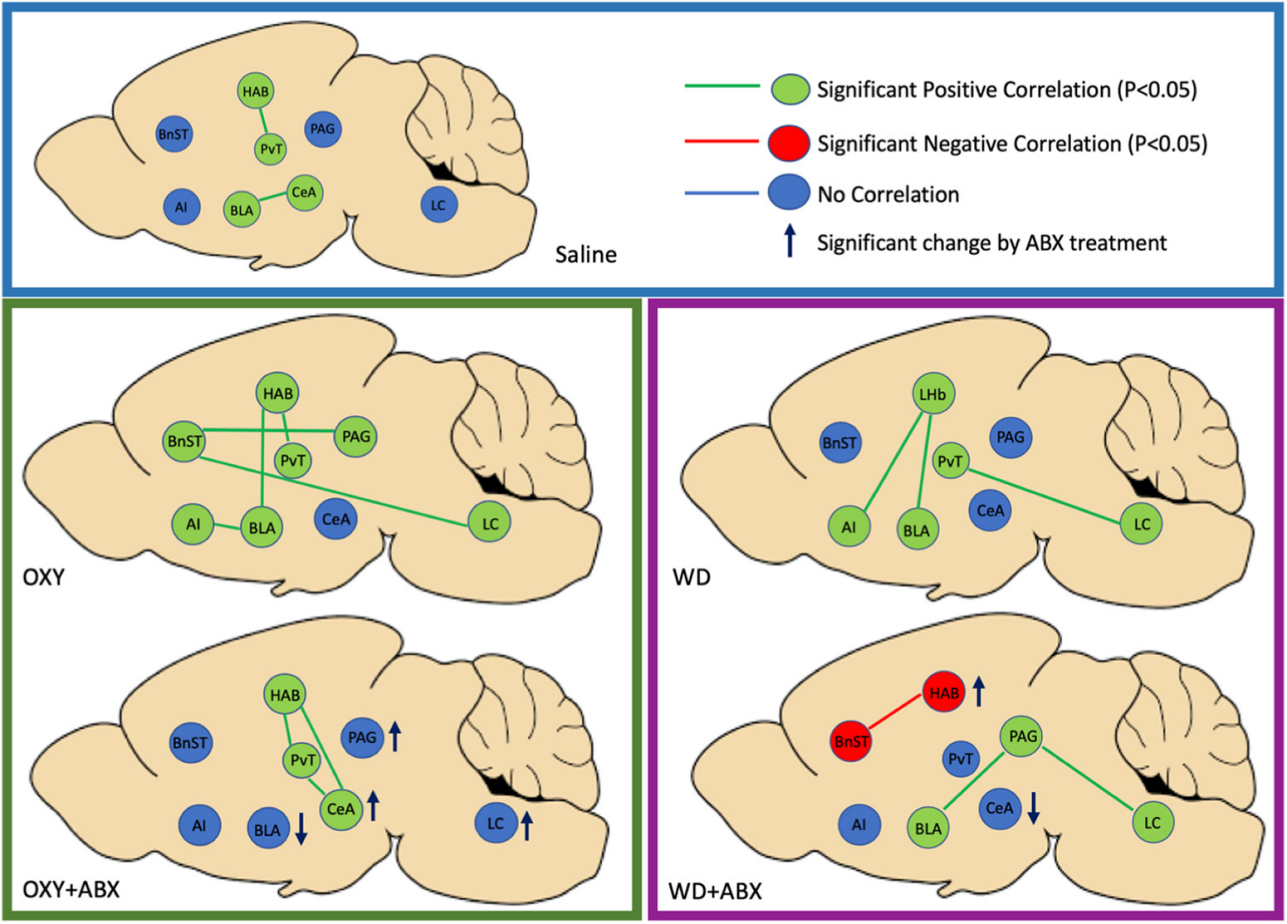
Correlational analysis of recruited neuronal ensembles. Depletion of the microbiome altered the recruitment of neuronal ensembles during oxycodone intoxication and withdrawal. Correlational analysis of Fos+ cells was performed between anatomically connected structures. Connections with significant correlations (*p *<* *0.05) are represented in green (positive) and red (negative). Nonsignificant correlations are shown in blue. The regions where recruited neurons significantly increased or decreased on antibiotic depletion are marked with blue arrows to indicate the direction of change.

We examined functional connectivity between brain regions based on significant correlations of Fos+ neurons. Saline control rats that were injected with naloxone exhibited significant positive correlations between the LHb and PVT (*R *=* *0.75, *p *<* *0.05) and between the BLA and CeA (*R *=* *0.78, *p *<* *0.05; Fig. 7). During intoxication, oxycodone-dependent rats exhibited an increase in the number of significant positive correlations (five correlations) compared with saline rats (two correlations). In the OXY group, the LHb was positively correlated with the PVT (*R *=* *0.78, *p *<* *0.05) and BLA (*R *=* *0.83, *p *<* *0.05). Compared with the SAL group, the OXY group did not exhibit a positive correlation between the BLA and CeA but exhibited a positive correlation between the BLA and AI (*R *=* *0.76, *p *<* *0.05). The BNST was not correlated with any other region in the SAL group. The BNST was positively correlated with the PAG (*R *=* *0.78, *p *<* *0.05) and LC (*R *=* *0.69, *p *<* *0.05) in the OXY group.

In the OXY+ABX group, an overall decrease in positively correlated regions was observed compared with the SAL and OXY groups. Similar to the SAL and OXY groups, the LHb and PVT were positively correlated in the OXY+ABX group (*R *=* *0.90, *p *<* *0.05). Unlike in the SAL and the OXY groups, the CeA was positively correlated with the LHb (*R *=* *0.90, *p *<* *0.05), BNST (*R *=* *0.76, *p *<* *0.05), and PVT (*R *=* *0.89, *p *<* *0.05) in the OXY+ABX group, which exhibited an overall increase in positive connectivity. The correlation between the BLA and CeA in the SAL group was not present in the OXY+ABX group. Depletion of the microbiome in the OXY+ABX group resulted in a decrease in the number of recruited Fos+ neurons in the BLA (−31%) compared with the matched OXY group. The PAG, CeA, and LC also exhibited 122%, 751%, and 119% increases, respectively, in the number of recruited Fos+ neurons in the OXY+ABX group compared with the OXY group.

### Microbiome depletion induced alterations of withdrawal network connectivity

During withdrawal, the WD group exhibited three positive correlations, contrasting to two positive correlations in the SAL group. In the WD group, the LHb was positively correlated with the BLA (*R *=* *0.61, *p *<* *0.05) and AI (*R *=* *0.62, *p *<* *0.05). The PVT was positively correlated with the LC in the WD group (*R* = 0.79, *p *<* *0.05). In contrast to the WD group, the WD+ABX group did not exhibit a positive correlation between the LHb and BLA or AI. The WD+ABX group exhibited a negative correlation between the LHb and BNST (*R* = −0.77, *p *<* *0.05). The LHb exhibited a 30% increase in the number of recruited Fos+ neurons, whereas the CeA exhibited a 40% decrease in the number of recruited Fos+ neurons in the WD+ABX group compared with the matched WD group. In the WD+ABX group, the PAG was positively correlated with the BLA (*R *=* *0.68, *p *<* *0.05) and LC (*R *=* *0.70, *p *<* *0.05), representing a shift in connectivity of the LC from the PVT to the PAG during withdrawal after microbiome depletion in the WD+ABX group.

## Discussion

The present study identified neuronal ensembles in the BLA, PAG, LC, and CeA that were altered by microbiome depletion during intoxication as well as ensembles in the CeA and LHb that were altered by microbiome depletion during withdrawal. Additionally, we provide evidence that microbiome depletion modulated the recruitment of neuronal ensembles in a state-specific and region-specific manner. The strongest effects were an increase in Fos activation in the PAG, LC, and CeA during intoxication and a decrease in Fos activation in the CeA during withdrawal. Furthermore, correlational analysis indicated that antibiotic depletion destabilized functional networks that were recruited during intoxication and withdrawal, with a decrease in correlation between brain regions.

The development of oxycodone dependence and state specificity was confirmed by von Frey pain thresholds and withdrawal scoring (Kononoff et al., 2018) after naloxone administration, which demonstrated the development of withdrawal-induced mechanical hyperalgesia and somatic signs of withdrawal. Naloxone-precipitated opioid withdrawal is robust, eliciting vigorous withdrawal symptoms in dependent animals (Gracy et al., 2001). We also identified several brain regions that were recruited during intoxication (BLA) and withdrawal (PAG, CeA, LC, PVT, AI, BNST, and LHb), further confirming that oxycodone produced neuroadaptations in key brain regions that are associated with addiction-like behaviors. During oxycodone intoxication, the only brain region that exhibited a significant increase in Fos+ neurons (688%) compared with saline controls was the BLA. The BLA sends projections to the nucleus accumbens (NAc) where it modulates behavioral conditioning and reward valence. This circuit has also been shown to alter operant behaviors through dopaminergic signaling (Wassum and Izquierdo, 2015). The BLA also has afferent and efferent connections with the CeA, which plays an integral role in anxiety, a distinct player in relapse and the escalation of intake (Tye et al., 2011). Alterations of either of these circuits could result in significant changes in the perception of reward or impact withdrawal symptoms, which could in turn initiate relapse and modify drug-taking behaviors.

Seven of eight regions of interest in the present study exhibited significantly higher counts of Fos+ neurons during withdrawal compared with controls. These regions (PAG, CeA, LC, PVT, AI, BNST, and LHb) have been shown to be associated with withdrawal-like behaviors, such as anxiety-like behavior, stress-related behaviors, pain, and negative emotional states in general (Koob, 2009), all of which are key components in the transition to dependence (Koob and Moal, 1997; Koob, 2008, 2009). For example, one region of the extended amygdala, the lateral BNST (Fig. 6), exhibited a 1272% increase in the number of Fos+ neurons in antibiotic-treated animals compared with the control group. This region highly expresses corticotrophin-releasing factor (CRF; Giardino et al., 2018) and has been implicated in anxiety-like behavior (Avery et al., 2016). Other regions that are involved in withdrawal, such as the PVT, LC, and LHb, exhibited 289%, 475%, and 712% increases, respectively, in the number of Fos+ neurons compared with the control group. These regions have also been shown to be involved in drug withdrawal (Ivanov and Aston, 2001; Haight and Flagel, 2014). The LC regulates arousal, responses to stress, and memory. Recruitment of the LC during withdrawal could result in an increase in the salience of each state (Ivanov and Aston, 2001; Van Bockstaele et al., 2010). The LHb is important for information processing and valence. This nucleus highly expresses tyrosine hydroxylase, which could result in aberrant dopamine signaling (Hikosaka et al., 2008, Aizawa et al., 2012; Proulx et al., 2014). These results confirmed the validity of the animal model that was used in the present study to produce opioid intoxication and dependence at both the behavioral and neurobiological levels.

No significant change in behavior was observed between antibiotic-depleted and non-depleted animals, suggesting that these behaviors in this paradigm did not depend on alterations of the microbiome or that we could not detect such changes because of either ceiling or floor effects during behavioral testing. Indeed, the severity of the somatic signs of withdrawal (1420% increase in withdrawal signs in the WD and WD+ABX groups) and hyperalgesia/allodynia (64% decrease in pain thresholds in the WD and WD+ABX groups) may have masked the contribution of the gut microbiome to these behaviors. Other studies reported that intermittent access to morphine in microbiome-depleted animals was associated with changes in hyperalgesia in the tail withdrawal test (Lee et al., 2018), but this was observed under conditions of 24-h spontaneous withdrawal. Moreover, the tail withdrawal test measures sensitivity to a thermal stimulus instead of mechanical allodynia. We also cannot exclude the possibility that the microbiome may be involved in other behaviors that are related to opioid dependence, including anxiety-like behavior, anhedonia-like behavior, or depression-like behavior. For example, a recent study reported that microbiome depletion exacerbated anxiety-like behavior, which could be rescued by the restoration of a healthy microbiome (van De Wouw et al., 2018). Further studies are required to test this hypothesis.

Further assessment of the microbiome after antibiotic treatment demonstrated a decrease in both Bacteroidetes and Firmicutes at the phylum level, thus demonstrating the efficacy of antibiotic treatment. The reductions of these phyla have been shown to result in a decrease in circulating short-chain fatty acids (i.e., metabolites that are released by the resident microbiota after the digestion of dietary fiber; Louis and Flint, 2017). These metabolites are capable of crossing the blood-brain barrier, signaling through a family of G-protein-coupled receptors (GPR41/GPR43; Kimura et al., 2011, 2014; Inoue et al., 2012), and acting as class II histone deacetylate inhibitors (Chen et al., 2003; Waldecker et al., 2008; Fellows et al., 2018), which in turn can alter gene expression profiles. The application of short-chain fatty acids has also been shown to reduce anxiety-like behaviors that are linked to relapse liability in addiction paradigms (van de Wouw et al., 2018; Baxley et al., 2019). Further exploration of the molecular mechanisms by which the microbiome alters the gut-brain axis are necessary to determine the role of microbiome alterations in drug-taking behaviors.

Despite the lack of changes at the behavioral level, the present results showed that antibiotic treatment produced robust changes in how the brain responded to oxycodone intoxication and withdrawal. Compared with the OXY group, the antibiotic-depleted OXY+ABX group exhibited alterations of the recruitment of four regions, including a 122% increase in the PAG, a 119% increase in LC, a 752% increase in the CeA, and a 31% decrease in the BLA. Interestingly, the PAG, LC, and CeA were highly recruited during withdrawal, whereas the BLA was recruited during intoxication (Fig. 3). These state-specific and region-specific changes in Fos+ neuron recruitment represented a major shift in the functional network of these regions, which was confirmed by the correlational analysis (Fig. 5).

The CeA is associated with negative emotional states and negative reinforcement during withdrawal (George et al., 2007; Koob, 2009; de Guglielmo et al., 2016), the activation of which decreased by 40% after antibiotic treatment. The CeA is known to be involved in withdrawal symptoms through the activation of CRF and GABA transmission (Funk et al., 2006; de Guglielmo et al., 2019). A significant increase or decrease in neuronal recruitment could result in the desynchronization of ensembles that regulate the perception of negative affective states (Iredale et al., 2000; Contarino and Papaleo, 2005; George et al., 2007). Another region that was affected by antibiotic treatment during withdrawal was the LHb, which exhibited a 30% increase in Fos+ neurons. The LHb has been implicated in the processing of emotional valence, depression, and withdrawal from drugs of abuse (Sartorius and Henn, 2007; Proulx et al., 2014; Shabel et al., 2014). These results indicate that the lack of behavioral changes after antibiotic treatment does not necessarily mean that the brain was not affected by microbiome depletion. Instead, despite the absence of behavioral alterations, we found that antibiotic treatment produced robust alterations of the sensitivity of multiple brain regions that are involved in oxycodone intoxication and withdrawal. This is an important result that further demonstrates the key role of the microbiome in the modulation of the brain response to drugs. Notably, we used a single marker of neuronal reactivity (Fos) that cannot capture the entirety of changes in functional brain networks. Therefore, additional changes would likely be observed with the use of alternative markers of neuronal reactivity (e.g., Arc and egr1).

To further assess the impact of antibiotic treatment on brain networks, we evaluated the degree of associations between changes in neuronal activity (i.e., functional connectivity) in each brain region during oxycodone intoxication and withdrawal (Kimbrough et al., 2020). We found that antibiotic treatment was associated with dysregulation of the functional network that was produced by decorrelations between several brain regions compared with the control groups. Among the brain regions where functional connections were perturbed, many express opioid peptides and receptors, such as the PAG, LHb, and LC. For example, the OXY group exhibited an overall increase in positive correlations between these brain regions, and these positive correlations were lost after microbiome depletion. A reduction of connectivity was also observed in the WD group, in which the positive correlation between the LHb and BLA was lost. In the PAG, the disruption of connectivity could lead to changes in pain levels, given its importance for pain processing (Chieng and Christie, 1996) and given that extended opioid use can alter pain thresholds (Chieng and Christie, 1996). During withdrawal, microbiome depletion also resulted in a shift in functional connectivity of the LC. In the WD group, the LC had a positive correlation with the PVT, whereas the LC had a positive correlation with the PAG in the WD+ABX group. Activation of the LC is modulated by opioid peptides (Kreibich et al., 2008), CRF, and excitatory amino acids and is well positioned to alter states of intoxication and withdrawal through both connectivity and signaling capability (Van Bockstaele et al., 1998). Overall, these results demonstrate that antibiotic treatment affected the number of Fos+ neurons that were recruited during opioid intoxication and withdrawal and affected the entire functional network of key brain regions that are involved in drug addiction.

Overall, the present study identified brain regions that were activated during oxycodone intoxication and withdrawal and provided evidence that antibiotic-induced depletion of the microbiome modulated the recruitment of neuronal ensembles in a state-specific and region-specific manner. The results showed that antibiotic treatment produced robust alterations of the sensitivity of multiple brain regions that are involved in oxycodone intoxication and oxycodone withdrawal, despite having no effects on addiction-related behaviors. This is an important result that further demonstrates the key role of the microbiome in the modulation of the brain response to drugs. Future studies that test drug self-administration and behaviorally characterize emotional states will provide alternative readouts of behavioral changes that are associated with microbiome depletion, including drug intake, compulsivity, and anxiety-like behavior. Further cellular and molecular characterizations of specific neuronal populations that are upregulated or downregulated following microbiome depletion and the identification of molecular targets that are modulated by microbiome metabolites are required to better understand the role of the gut-brain axis in addiction and identify possible new treatment targets.

## Synthesis

Reviewing Editor: Karen Szumlinski, University of California at Santa Barbara Decisions are customarily a result of the Reviewing Editor and the peer reviewers coming together and discussing their recommendations until a consensus is reached. When revisions are invited, a fact-based synthesis statement explaining their decision and outlining what is needed to prepare a revision will be listed below. The following reviewer(s) agreed to reveal their identity: Wendy Walwyn.

The authors have addressed the prior concerns of the reviewers.
